# Outstanding increase in tumor-to-background ratio over time allows tumor localization by [^89^Zr]Zr-PSMA-617 PET/CT in early biochemical recurrence of prostate cancer

**DOI:** 10.1186/s40644-024-00778-5

**Published:** 2024-10-07

**Authors:** Caroline Burgard, Florian Rosar, Elena Larsen, Fadi Khreish, Johannes Linxweiler, Robert J. Marlowe, Andrea Schaefer-Schuler, Stephan Maus, Sven Petto, Mark Bartholomä, Samer Ezziddin

**Affiliations:** 1 Departments of Nuclear Medicine, Saarland University – Medical Center, Kirrberger Str. 100, Geb. 50, D-66421 Homburg, Germany; 2 Departments of Urology, Saarland University – Medical Center, Homburg, Germany; 3 Spencer-Fontayne Corporation, Jersey City, NJ USA; 4 Present Address: Department of Nuclear Medicine, Klinikum Fulda gAG, Fulda, Germany; 5 Saarland University, USAAR, Saarbrücken, Germany

**Keywords:** Biochemical recurrence, Localization, Positron emission tomography/Computed tomography (PET/CT), Prostate cancer, Prostate-specific membrane antigen (PSMA), Zirconium-89 (^89^Zr)

## Abstract

**Background:**

Positron emission tomography/computed tomography (PET/CT) using prostate-specific membrane antigen (PSMA)-targeted radiotracers labeled with zirconium-89 (^89^Zr; half-life ~ 78.41 h) showed promise in localizing biochemical recurrence of prostate cancer (BCR) in pilot studies.

**Methods:**

Retrospective analysis of 38 consecutive men with BCR (median [minimum–maximum] prostate-specific antigen 0.52 (0.12–2.50 ng/mL) undergoing [^89^Zr]Zr-PSMA-617 PET/CT post-negative [^68^Ga]Ga-PSMA-11 PET/CT. PET/CT acquisition 1-h, 24-h, and 48-h post-injection of a median (minimum–maximum) [^89^Zr]Zr-PSMA-617 tracer activity of 123 (84–166) MBq.

**Results:**

[^89^Zr]Zr-PSMA-617 PET/CT detected altogether 57 lesions: 18 local recurrences, 33 lymph node metastases, 6 bone metastases in 30/38 men with BCR (78%) and prior negative conventional PSMA PET/CT. Lesion uptake significantly increased from 1-h to 24-h and, in a majority of cases, from 24-h to 48-h. Tumor-to-background ratios significantly increased over time, with absolute increases of 100 or more. No side effects were noted. After [^89^Zr]Zr-PSMA-617 PET/CT-based treatment, prostate-specific antigen concentration decreased in all patients, becoming undetectable in a third of patients. Limitations: retrospective, single center design; infrequent histopathological and imaging verification.

**Conclusion:**

This large series provides further evidence that [^89^Zr]Zr-PSMA-617 PET/CT is a beneficial imaging modality to localize early BCR. A remarkable increase in tumor-to-background ratio over time allows localization of tumor unidentified on conventional PSMA PET/CT.

## Background

In men with biochemical recurrence of prostate cancer (BCR), identifying the location and extent of disease is essential for optimizing treatment plans [1]. Presently, the preferred procedure for this purpose is prostate-specific membrane antigen (PSMA)-targeted positron emission tomography/computed tomography (PET/CT). Current tracers rely on the short-lived radionuclides gallium-68 (^68^Ga; half-life: ~67.7 min) or fluorine-18 (^18^F, half-life ~ 110.8 min) [2–9]. With these tracers, lesions with only weak PSMA expression and slowly increasing uptake may not or only insufficiently be visualized [10–16]. Additionally, the requirement that images be obtained relatively shortly post-administration limits time for radiotracer clearance from non-target tissue, potentially contributing to an insufficient tumor-to-background ratio, i.e., poor contrast [16].

Use of tracers incorporating zirconium-89 (^89^Zr) may represent a promising avenue to address these limitations of radiopharmaceuticals with short-lived radionuclides. The relatively protracted half-life of ^89^Zr, ~ 78.41 h, permits much later imaging than is possible with ^68^Ga or ^18^F, namely, scanning at ≥ 24-h post-injection. We and others have demonstrated in single cases and small series of patients with BCR that PET/CT at ≥ 24-h post-injection with different ^89^Zr-labeled PSMA-targeted radiotracers frequently reveals lesions suspicious for prostate cancer that were undetected with conventional PSMA-targeted PET/CT [10–16]. Our group also has used ^89^Zr-based late scanning to provide insight into the suspiciousness of indeterminate findings on conventional PSMA-targeted scans [12, 17].

Our encouraging preliminary results led us to retrospectively analyze the use of [^89^Zr]Zr-PSMA-617 PET/CT in a larger sample of patients with BCR and recent prior negative [^68^Ga]Ga-PSMA-11 PET/CT. Our goals were to further verify the detection efficacy of [^89^Zr]Zr-PSMA-617 PET/CT, and to gather more data on the lesion uptake kinetics of the novel tracer.

## Methods

### Study design and endpoints

The primary endpoint of this study was to determine the detection efficiency of [^89^Zr]Zr-PSMA-617 PET/CT with respect to suspicious lesions in a larger cohort of patients with BCR and negative conventional PSMA PET/CT.

Secondary endpoints were the magnitude of lesion uptake of [^89^Zr]Zr-PSMA-617 and contrast to background over time, as well as a comparison of values of related PET variables with each other and with those of [^68^Ga]Ga-PSMA-11 in the conventional scan for sites of positive [^89^Zr]Zr-PSMA-617 PET/CT findings. The remaining secondary endpoints were near-term safety of [^89^Zr]Zr-PSMA-617 PET/CT, i.e. adverse events or vital sign abnormalities observed during or shortly after the procedure that we considered to be related to [^89^Zr]Zr-PSMA-617 PET/CT and the follow-up after [^89^Zr]Zr-PSMA-617 PET/CT-based treatment (e.g. radiotherapy or surgical resection of the metastasis) including serum PSA levels and imaging (e.g. follow up PSMA PET/CT).

### Patients and ethics

The cohort comprised 38 consecutive men with BCR, defined as increasing prostate-specific antigen (PSA) following primary (curative-intent) treatment, who from 25 October 2021–8 May 2023, underwent [^68^Ga]Ga-PSMA-11 PET/CT with negative findings, and then, within a short period [^89^Zr]Zr-PSMA-617 PET/CT. All imaging took place at Saarland University Medical Center. To be included, patients also had to have unaltered prostate cancer treatment during the interval between the conventional scan and the experimental scan. None of the patients showed clinical progression between [^68^Ga]Ga-PSMA-11 or [^89^Zr]Zr-PSMA-617 PET/CT.

Table 1 summarizes patient and imaging characteristics of the study sample. This cohort was middle-aged to elderly, with Gleason stage 7 disease in approximately 2/3. The PSA levels obtained at the time of [^89^Zr]Zr-PSMA-617 PET/CT were low (median [minimum–maximum] 0.52 [0.11–2.50] ng/mL). Primary treatment included prostatectomy in all patients. The analysis conformed to the Declaration of Helsinki and received ethics approval from the Institutional Review Board of the Ärztekammer des Saarlandes/Saarbrücken (approval number: 170/22, 13 September 2022). All patients provided written informed consent for [^89^Zr]Zr-PSMA-617 PET/CT and allowed publication of de-identified patient data.

**Table 1:** Patient and imaging characteristics of 38 consecutive men with BCR and negative recent prior [^68^Ga]Ga-PSMA-11 PET/CT

Characteristic	Value
Age [yr]	
Median (min.–max.)	69 (53–82)
PSA [ng/mL], median (min.-max.)	
At [^68^Ga]Ga-PSMA-11 PET/CT	0.47 (0.08–2.49)
At [^89^Zr]Zr-PSMA-617 PET/CT	0.52 (0.11–2.50)
PSA doubling time category, % (n)	
<3 mo.	21% (8)
3–6 mo.	26% (10)
7–12 mo.	18% (7)
>12 mo.	34% (13)
Gleason Score category, % (n)	
6	5% (2)
7a	26% (10)
7b	34% (13)
8	18% (7)
9	16% (6)
Primary treatment, % (n)	
Prostatectomy	100% (38)
Additional salvage treatments% (n)	
Radiation therapy	34% (13)
ADT	18% (7)
Lymphadenectomy	5% (2)

Because of rounding, percentages may not add up to 100% for certain characteristics

ADT, androgen deprivation therapy; max., maximum; min., minimum; PSA, prostate-specific antigen; SD, standard deviation

### [^68^Ga]Ga-PSMA-11 PET/CT

[^68^Ga]Ga-PSMA-11 scans were acquired using a median (minimum–maximum) 148.5 (111–184) MBq of radiotracer. PET/CT was performed ~ 1 h post-infusion, following standard procedures [18]. Findings were classified visually by consensus among three nuclear medicine physicians who were experienced PET/CT readers (SE, FK, FR); medical histories and prior images were available to aid interpretation. Negative scans were defined as those lacking pathological uptake.

### [^89^Zr]Zr-PSMA-617 PET/CT

[^89^Zr]Zr-PSMA-617 PET/CT took place a median (minimum–maximum) 38 (5–126) d after [^68^Ga]Ga-PSMA-11 PET/CT. Scans with the novel tracer were performed 1-h, 24-h, and 48-h post-administration. The 1-h scan was acquired to allow direct comparison with the prior conventional scan. The 24-h and 48-h scans evaluated imaging that would be expected to yield appropriate images with the long-lived radionuclide. After intravenous infusion of a median (minimum–maximum) 123 (84–166) MBq of [^89^Zr]Zr-PSMA-617, immediately followed by 500 mL of NaCl 0.9%, whole-body PET/CT images, extending from the vertex to the mid-femur, were obtained using a Biograph mCT 40 system (Siemens Medical Solutions, Knoxville, TN, USA). PET acquisition time was 3 min/bed position for the 1-h scan, 4 min/bed position for the 24-h scan, and 5 min/bed position for the 48-h scan. For attenuation correction and anatomical localization, low-dose CT was performed at a 120-keV x-ray tube voltage. Tube current modulation with CARE Dose4D software (Siemens Healthineers, Erlangen, Germany) was employed, with 30 mAs as the reference. A soft tissue kernel (B31f/Be32) and a slice thickness of 5 mm (increment: 2–4 mm) were used to reconstruct data, which also were corrected for decay, randoms, and scatter. To reconstruct PET images, an iterative 3-dimensional ordered-subset expectation maximization algorithm (3 iterations; 24 subsets) was applied, and Gaussian filtering was carried out to a transaxial resolution of 5 mm at full width at half maximum. Respective matrix and pixel sizes were 200 × 200 and 3.0 mm. [^89^Zr]Zr-PSMA-617 PET/CT was performed on a compassionate use basis per the German Pharmaceutical Act § 13 (2b). Attending nuclear medicine specialists oversaw the procedure, including requisitioning the radiopharmaceutical, which was manufactured in-house [12].

### [^89^Zr]Zr-PSMA-617 PET/CT image analysis

[^89^Zr]Zr-PSMA-617 PET/CT findings also were classified visually by consensus, by the same readers as with the conventional scans. Again, since interpretation was made within everyday practice, medical history and prior images were accessible. Lesions were deemed to be suspicious for prostate cancer if they were discernible on the 24-h scan and/or the 48-h scan in typical sites of pathologic uptake of PSMA-targeted tracers. Scans were deemed to be negative if no pathologic foci were seen.

For each scan (1-h, 24-h, 48-h post-injection), key PET variables reflecting radiotracer uptake and contrast were determined for each suspicious lesion on [^89^Zr]Zr-PSMA-617 PET/CT. To assess uptake, the maximum standardized uptake value (SUV_max_) was measured using SyngoVia Enterprise VB 60 software (Siemens Healthineers, Erlangen, Germany). For comparison, uptake was measured at the corresponding site in the early imaging of [^89^Zr]Zr-PSMA-617 and [^68^Ga]Ga-PSMA-11 PET/CT, even though no lesion could be clearly identified at this site.

To characterize contrast, the tumor-to-muscle ratio (TMR), tumor-to-liver ratio (TLR), tumor-to-contralateral region ratio (TCR), and tumor-to-background ratio (TBR) were calculated. These variables were defined as the SUV_max_ of the lesion divided by the mean SUV (SUV_mean_) of the respective comparator. SUV_mean_ was determined in spherical volumes-of-interest in the comparator location, i.e., gluteal muscle for TMR, central liver for TLR, the contralateral region for TCR, and the directly surrounding region for TBR.

## Statistics

Data are presented as descriptive statistics including, as appropriate, median (minimum–maximum), mean ± standard deviation (SD), and number (percentage) or vice versa. To compare uptake and contrast, Wilcoxon signed-rank testing was applied, using Prism version 9.0.0 (GraphPad Software, San Diego, CA, USA). A p value ≤ 0.05 was considered to be significant.

### Monitoring for potential adverse events

Adverse events and material vital signs abnormalities reported by health care professionals, patients, or both during imaging and up to ~ 4wk afterwards were documented. During the first visit post-imaging, and/or in telephone calls made shortly post-discharge, patients were queried regarding their experiences of specific side effects and, in open-ended fashion, regarding side effects in general.

## Follow-up

Follow-up data were acquired from July 2022 until April 2024. For those patients who underwent a treatment after [^89^Zr]Zr-PSMA-617 PET/CT, we collected PSA levels and analyzed their change from the time of experimental scanning. We compared biochemical response in subgroups based on their type of therapy. Where available, post-treatment imaging and histopathology, including immunohistochemistry, also were analyzed.

## Results

### **[**^**89**^**Zr]Zr-PSMA-617 PET/CT visual findings**

Lesions suspicious for prostate cancer were discernible in [^89^Zr]Zr-PSMA-617 PET/CT in 30 of 38 patients (78%); 8 men (22%) had consistently negative imaging (Table 2). PSA ranged from 0.13 ng/mL to 2.5 ng/mL (median 0.52 ng/mL) in men with positive imaging, and 0.11 ng/mL to 1.33 ng/mL (median 0.53 ng/mL) in those with negative findings.

In total, 57 lesions (minimum–maximum 1–4/patient) were visualized in patients with positive [^89^Zr]Zr-PSMA-617 PET/CT. Sixteen patients had only one finding (10 in prostate bed, 4 in lymph nodes, 2 in the skeleton), 7 had 2 findings, 5 had 3, and 2 had 4 lesions.

**Fig. 1:**
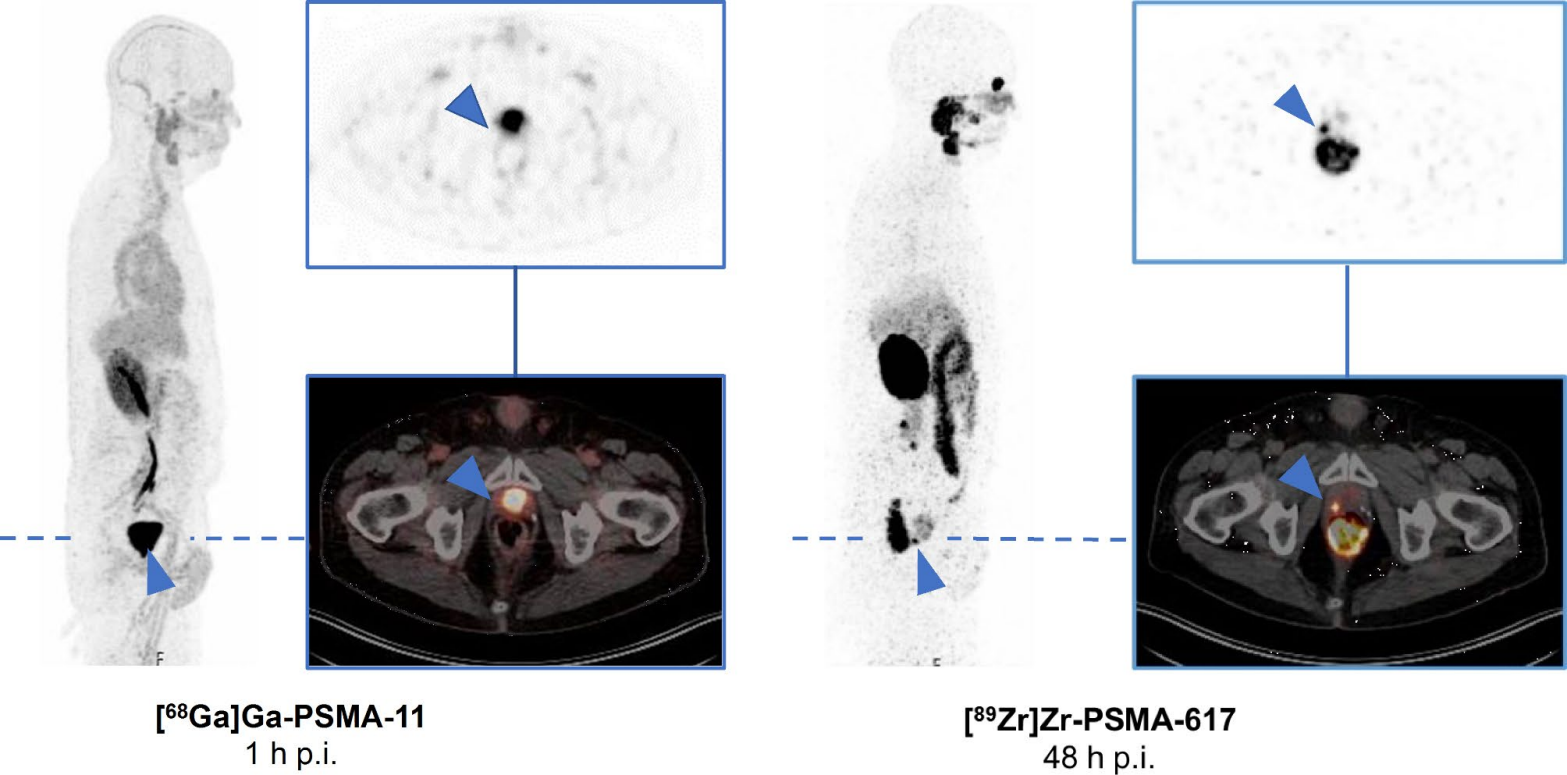
Maximum intensity projection (MIP) and transversal slices from a 48-h post-injection (p.i.) [^89^Zr]Zr-PSMA-617 PET/CT scan of a BCR patient (PSA level 0.28 ng/mL; gleason score 7a) that was positive for a suspicious lesion in the right former prostate bed (blue arrow), compared with corresponding prior negative 1-h p.i. [^68^Ga]Ga-PSMA-11 PET/CT images (PSA level 0.20 ng/mL; 82 days between both PET/CT). The patient received a [^89^Zr]Zr-PSMA-617-guided radiotherapy with a follow-up PSA level becoming undetectable (< 0.07 ng/mL)

**Fig. 2:**
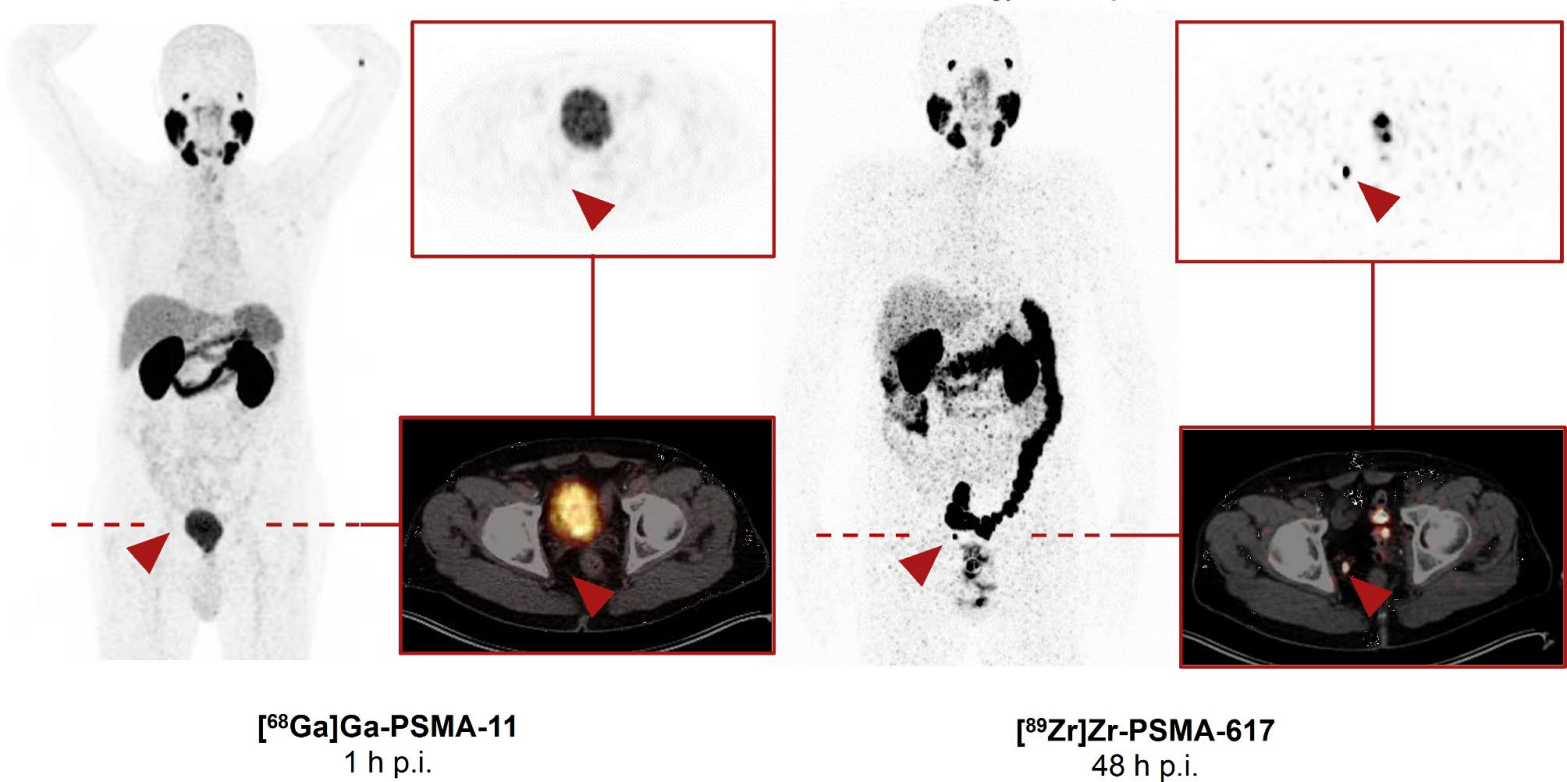
Maximum intensity projection (MIP) and transversal slices from a 48-h p.i. [^89^Zr]Zr-PSMA-617 PET/CT scan of a BCR patient (PSA level 2.50 ng/mL; gleason score 7a) that was positive for a suspicious lymph node lesion (red arrow), compared with corresponding prior negative 1-h p.i. [^68^Ga]Ga-PSMA-11 PET/CT images (PSA level 2.49 ng/mL; 13 days between both PET/CT). The patient received a [^89^Zr]Zr-PSMA-617-guided radiotherapy with a follow-up PSA level of 0.19 ng/mL

**Fig. 3:**
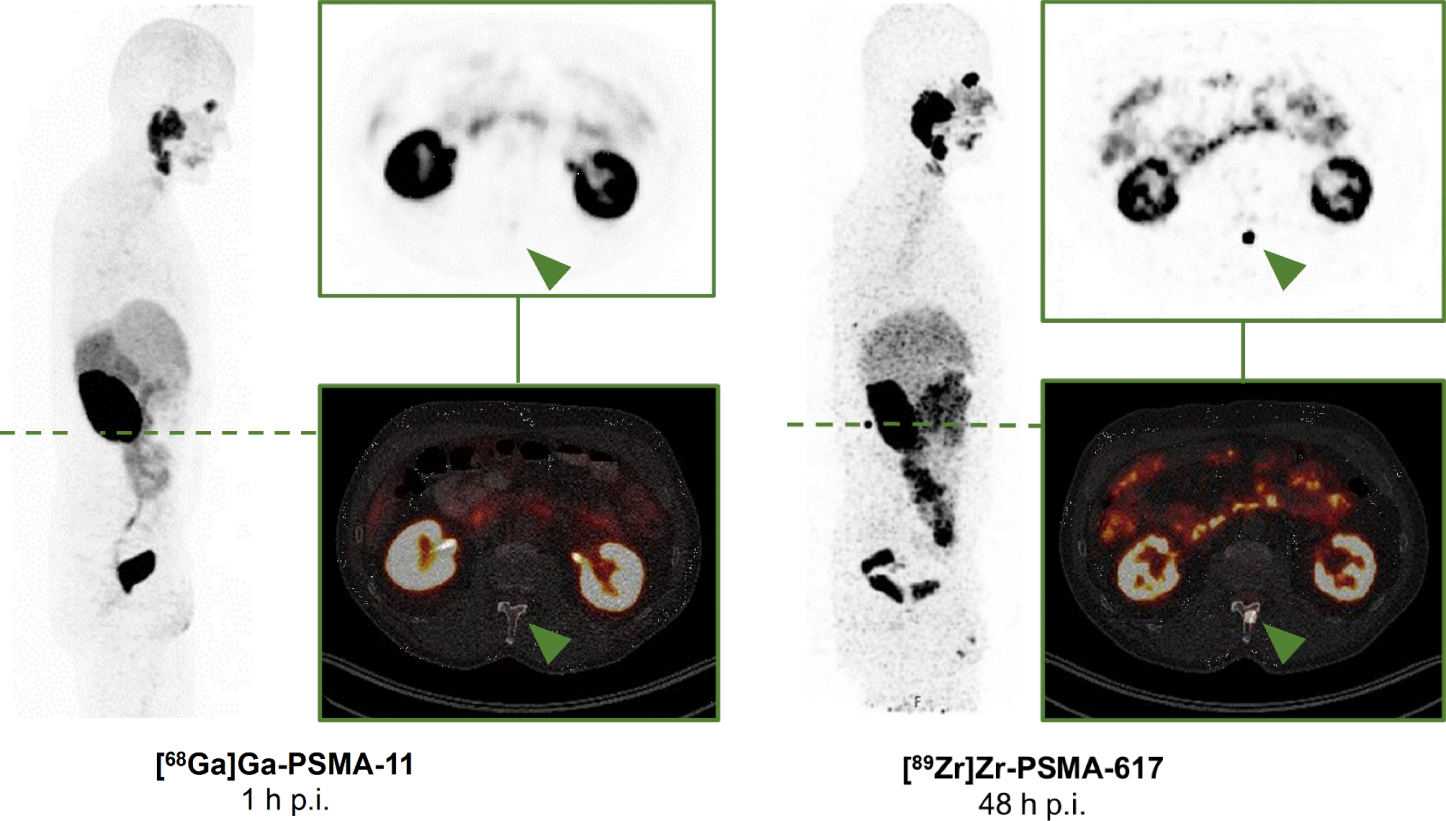
MIP and transversal slices from a 48-h p.i. [^89^Zr]Zr-PSMA-617 PET/CT scan of a BCR patient (PSA level 0.85 ng/mL; gleason score 9) that was positive for a suspicious bone lesion (green arrow), compared with corresponding prior negative 1-h p.i. [^68^Ga]Ga-PSMA-11 PET/CT images (PSA level 0.72 ng/mL; 34 days between both PET/CT). Subsequent magnet resonance imaging confirmed the suspicious bone lesion. The patient received a [^89^Zr]Zr-PSMA-617-guided radiotherapy with a follow-up PSA level becoming undetectable (< 0.07 ng/mL)

Of the 57 foci, 18 were seen in the prostate bed, 33 in lymph nodes, and 6 in the skeleton. Representative 48-h [^89^Zr]Zr-PSMA-617 PET/CT images of each type of lesion are compared with corresponding (1-h) [^68^Ga]Ga-PSMA-11 PET/CT images of the same sites in Figs. 1, 2 and 3.

### PET variables

#### [^89^Zr]Zr-PSMA-617

**Fig. 4:**
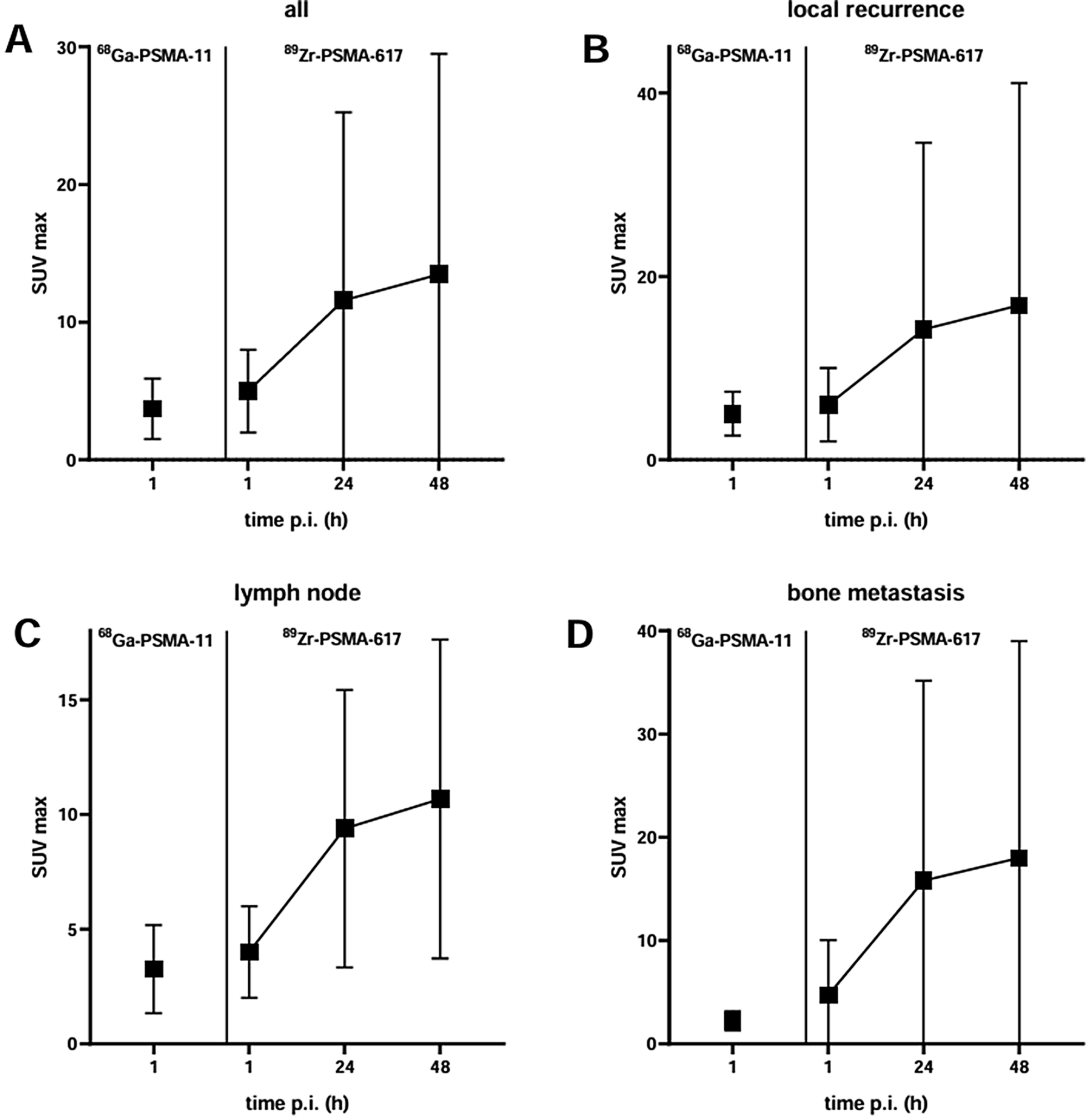
SUV_max_ (mean ± SD) of (**A**) all suspicious lesions, referred as (**B**) local recurrence (**C**) lymph node metastasis, and (**D**) bone metastasis by scan type and scan time

Statistics of SUV_max_ in suspicious lesions over time are presented in Fig. 4a. For all lesions, SUV_max_ increased markedly from 1-h to 24-h imaging (1 h, 5.1 ± 3.3; 24 h, 11.6 ± 13.7) and slightly further from 24-h to 48-h imaging (24 h, 11.6 ± 13.7, 48 h, 13.5 ± 16.0). Both increases were significant (*p* < 0.0001 and *p* = 0.0021). Figure 4b–d shows SUV_max_ values over time for all lesion categories (former prostate bed, lymph node and bone).

**Fig. 5:**
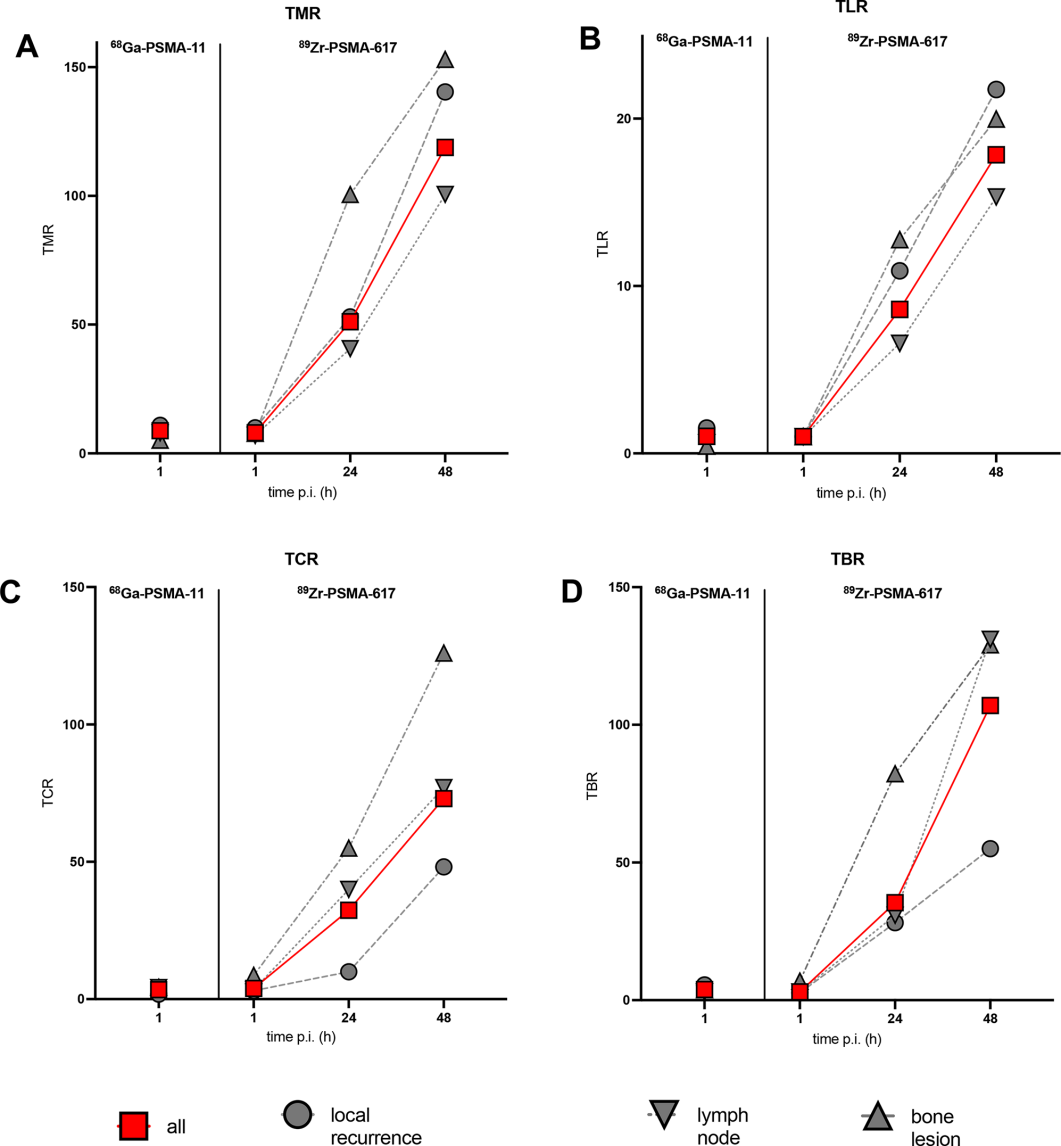
Mean lesion contrast, i.e., (**A**) TMR, (**B**) TLR, (**C**) TCR, and (**D**) TBR by scan type, scan time, and lesion type

In distinction with SUV_max_ findings, all four variables reflecting lesion contrast, namely TMR, TLR, TCR, and TBR, were significantly greater on average in 24-h versus 1-h images, as well as in 48-h versus 24-h images (all *p* ≤ 0.0001), with some increases exceeding 100 over time (Fig. 5).

### Comparison with [^68^Ga]Ga-PSMA-11

A slight but significant difference in SUV_max_ was observed between the prior [^68^Ga]Ga-PSMA-11 PET/CT scan and the 1-h [^89^Zr]Zr-PSMA-617 PET/CT scan (3.7 ± 2.2 versus 5.1 ± 3.3, *p* < 0.0001). This pattern also was seen for TLR (*p* < 0.0001), but not for TMR, TCR, or TBR (all *p* > 0.18). Detailed values of SUV_max_ and lesion contrast are summarized in Table 2.

**Table 2:** SUV_max_ and lesion contrast (TMR, TLR, TCR, and TBR) variables

	*[* ^*68*^ *Ga-PSMA-11 PET/CT*	*[* ^*89*^ *Zr]Zr-PSMA-617n PET/CT*			
	*1-h scan*	*1-h scan*	*24-h scan*	*48-h scan*	*p*
SUV_max_					
All lesions	3.7 ± 2.2	5.1 ± 3.3	11.6 ± 13.7	13.5 ± 16.0	0.0001, < 0.0001, 0.0021
Local recurrence	5.0 ± 2.4	6.3 ± 4	14.2 ± 20.3	16.8 ± 24.2	0.0396, 0.0987, 0.12
Lymph node metastasis	3.2 ± 1.9	4.5 ± 2.3	9.4 ± 6	10.6 ± 6.9	0.0049, < 0.0001, 0.07
Bone metastasis	2.2 ± 0.9	4.7 ± 5.3	15.8 ± 19.4	18.8 ± 21	0.19, 0.0312, 0.0312
TMR					
All lesions	8.6 ± 4.6	8.3 ± 5.3	51.1 ± 51.5	118.8 ± 144	0.18, **< 0.0001**,** 0.0001**
Local recurrence	10.9 ± 4.8	9.9 ± 6.6	53.8 ± 70.6	140.4 ± 179.7	0.20, **< 0.0001**,** 0.0003**
Lymph node metastasis	7.9 ± 4.2	7.5 ± 3.8	40.6 ± 27.1	100.6 ± 122.8	0.35, **< 0.0001**,** 0.0019**
Bone metastasis	5.1 ± 1.9	7.7 ± 7.9	100.5 ± 65.8	153.9 ± 143.6	> 0.99, **0.0312**, 0.56
TLR					
All lesions	1.0 ± 1.1	1.5 ± 1	8.6 ± 9.1	17.8 ± 17.7	**< 0.0001**,** 0.0001**,** < 0.0001**
Local recurrence	0.9 ± 0.5	1.9 ± 1.3	10.9 ± 13.4	21.7 ± 26.9	**0.0432**,**< 0.0001 < 0.0001**
Lymph node metastasis	0.6 ± 0.4	1.4 ± 0.6	6.6 ± 4.3	15.3 ± 9.4	**< 0.0001**,**< 0.0001< 0.0001**
Bone metastasis	0.4 ± 0.1	1.4 ± 1.7	12.7 ± 11.7	19.9 ± 20.2	0.0312, **0.0312**, 0.22
TCR					
All lesions	3.5 ± 3.9	3.9 ± 4.9	32.4 ± 49.2	73.6 ± 105.8	0.66, **< 0.0001**,** 0.0001**
Local recurrence	1.8 ± 1.1	3.1 ± 4.9	10.9 ± 17.7	48.1 ± 102.7	0.32, **0.0003**,** 0.0001**
Lymph node metastasis	4.2 ± 4.6	3.5 ± 2.3	39.9 ± 58.4	77.9 ± 87.2	0.62, **< 0.0001**,** < 0.0001**
Bone metastasis	4.3 ± 3.7	8.6 ± 11	54.9 ± 40.1	126.5 ± 187.3	0.44, **0.0312**, 0.31
TBR					
All lesions	3.9 ± 3.8	3.4 ± 2.7	35.4 ± 50.7	107 ± 129.8	0.99, **< 0.0001**,** 0.0001**
Local recurrence	5.5 ± 6.1	2.7 ± 1.6	28.1 ± 45	55.3 ± 42	0.2462, **< 0.0001**,** 0.0047**
Lymph node metastasis	3.0 ± 1.5	3.1 ± 1.8	30.8 ± 38.5	131.1 ± 25.8	1.00, **< 0.0001**,** < 0.0001**
Bone metastasis	3.7 ± 2.7	6.9 ± 5.9	82.2 ± 97.5	129 ± 167.4	0.06, **0.0312**, 0.16

p values in bold type were statistically significant at *p* ≤ 0.05

SD, standard deviation; SUV_max_, maximum standardized uptake value; TBR, tumor-to-background ratio; TLR, tumor-to-liver ratio; TMR, tumor-to-gluteal-muscle ratio; TCR, tumor-to-contralateral tissue ratio

### Safety

No adverse events, including material vital signs abnormalities, that were deemed to be related to [^89^Zr]Zr-PSMA-617 PET/CT were noted during the procedure or the ~ 4 weeks thereafter.

### Follow-up

**Fig. 6:**
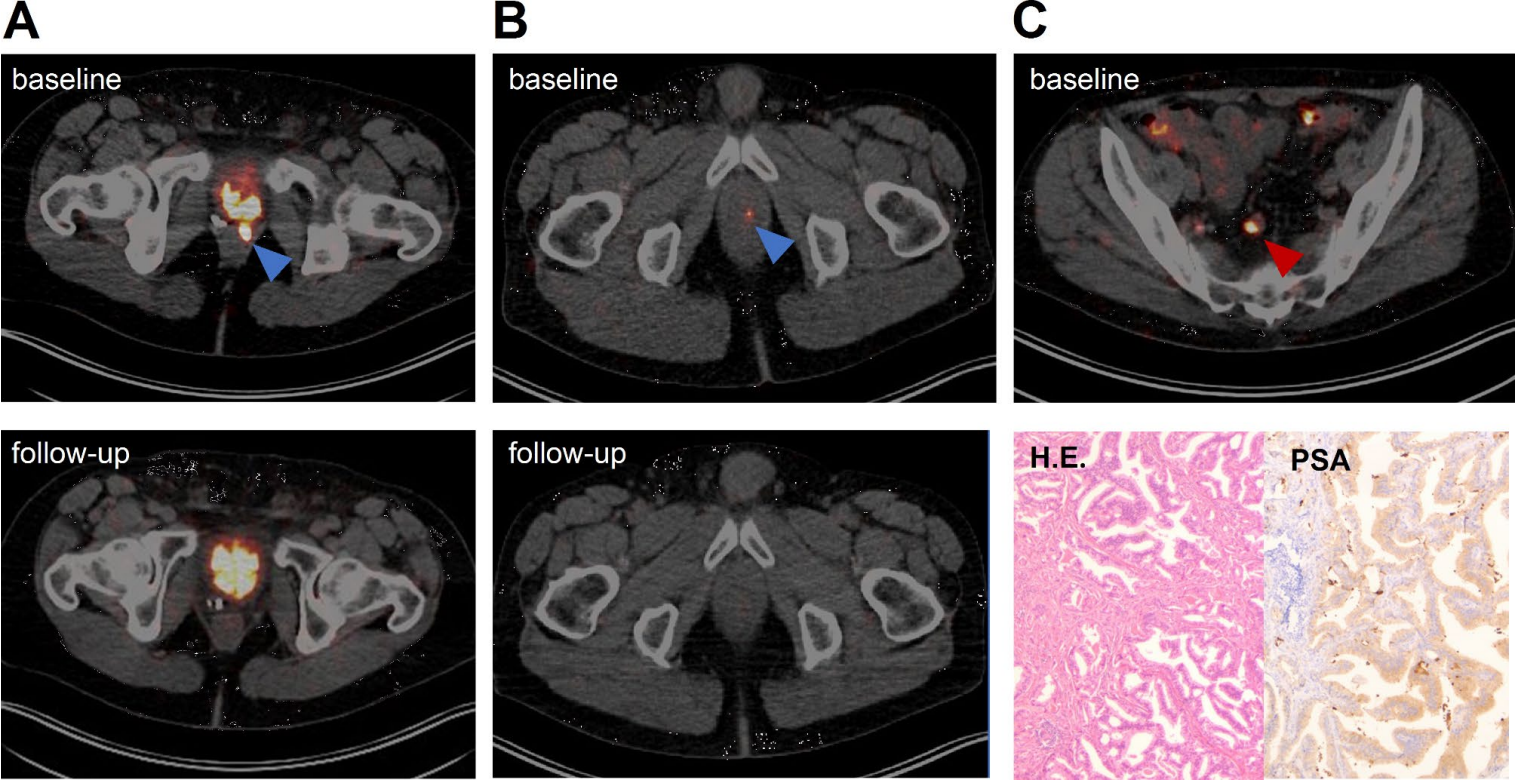
**A**,** B** Transversal slices from a 48-h p.i. [^89^Zr]Zr-PSMA-617 PET/CT scan that was positive for local recurrence (blue arrow), compared with a 48-h p.i. [^89^Zr]Zr-PSMA-617 PET/CT follow-up scan after radiation therapy, respectively. **C** Transversal slice from a 48-h p.i. [^89^Zr]Zr-PSMA-617 PET/CT scan that was positive for a suspicious lymph node lesion (red arrow), with post-surgical histological work-up with H.E.- and PSA staining confirming prostate cancer metastasis

After positive findings on [^89^Zr]Zr-PSMA-617 PET/CT, 29 of 30 patients started treatment, including PET-guided radiation therapy, surgical intervention, or androgen deprivation therapy (ADT). One patient (3%, with prior history of radiotherapy) chose to stay on active surveillance. In 24/30 men (80%, 3 with prior history of radiotherapy, 3 with ADT), [^89^Zr]Zr-PSMA-617-guided radiotherapy was performed and all these patients’ PSA levels decreased (mean decrease: 82% ± 28%), becoming undetectable (< 0.07 ng/mL) in 10 patients. In total, 14 suspicious lesions in 7 patients were radiographically confirmed by [^89^Zr]Zr-PSMA-617 PET/CT after local therapy. Examples of follow-up imaging after local therapy are depicted in Fig. 6. In 4 patients (13%, 2 with prior history of radiotherapy), ADT was initiated, and afterwards, their PSA levels decreased by a mean 92% ± 7%. One patient (3%, with prior history of radiotherapy) underwent lymphadenectomy with a subsequent PSA decrease of 90%. Histopathological workup confirmed prostate cancer metastasis in this case (Fig. 6C).

## Discussion

Here we report an analysis from a 38-patient cohort regarding the ability of [^89^Zr]Zr-PSMA-617 PET/CT to effectively localize prostate cancer in men with early BCR in whom prior conventional imaging with [^68^Ga]Ga-PSMA-11 failed to detect the culprit lesion(s). We capitalized on favorable properties of this [^89^Zr]-labelled PSMA-targetedradiotracer, i.e., ability to attain delineating uptake in prostate cancer lesions with a remarkable increase in contrast in late scans.

This analysis, to our knowledge the largest yet published of PET/CT using a ^89^Zr-labelled PSMA-targeted radiotracer in the BCR setting, confirms and extends efficacy results in that setting that were previously reported by our group and others [11–13, 15, 16]. Our patient-level detection rate of 79% (30/38) aligns with that previously reported by our team in subgroups of the present cohort (78%, 18/23) [16]. In addition, our detection rate appreciably exceeded the high rate reported by Dietlein et al., 57% (8/14), using a different radiotracer, ^89^Zr-PSMA-Df, at 24–72-h post-injection in a cohort with prior negative conventional scanning [14]. Our analysis reveals a dramatic increase of uptake, especially lesion contrast over time when compared to the standard conventional imaging timepoint. This increase explains the high detection rate of [^89^Zr]Zr-PSMA-617 PET/CT in recurrence not detected by conventional PET imaging and demonstrates the superiority of PSMA-PET/CT with long-lived PET tracers, which allow for delayed imaging after injection. This applies to local recurrences, lymph node metastases as well as distant metastases.

The clinical relevance of successful localization of otherwise undetected lesions/disease is strongly implied by the experience of our patients with positive [^89^Zr]Zr-PSMA-617 scans and subsequently successful local therapy (mainly imaging-guided radiation) or a switch from local to systemic therapy because of previously occult multifocal / disseminated disease. Further follow-up in a larger cohort should be able to additionally demonstrate the outcome benefits from optimized treatment based on successful localization of previously undetected disease. These benefits could include longer PSA recurrence-free survival resulting from PET-targeted/adapted radiation therapy protocols, or the application of metastasis-directed therapy in general, often only possible, or at least facilitated, by accurate detection of disease site and extent. In our cohort, the clinical course following ^89^Zr-based imaging and subsequent local or systemic therapy strongly suggested that findings seen on PET/CT with ^89^Zr-labeled radiopharmaceuticals indeed correspond to prostate cancer, regarding the results of biochemical, imaging and histopathological follow-up.

The present work also confirms the near-term safety of [^89^Zr]Zr-PSMA-617 PET/CT. There were no apparent side effects of the procedure during scanning or the weeks thereafter. Nonetheless, additional, prospective, and longer-term safety observations would be desirable. In the meanwhile, the lack of longer-term side effects in the literature to date regarding patients with prostate cancer, and the good safety and tolerability of zirconium-labeled radiopharmaceuticals in other settings [19] support our safety findings.

Besides infrequent histopathological confirmation and lack of long-term safety data, our analysis had the limitations of being retrospective, observational, and single center. These characteristics reduce strength-of-evidence and generalizability. Multicenter, prospective studies of this imaging modality should be undertaken. Another limitation is the interval of up to 126 days between the [^68^Ga]Ga-PSMA-11 PET/CT and the [^89^Zr]Zr-PSMA-617 PET/CT. During this period, it cannot be ruled out that previously unidentified lesions would not have been faintly or clearly visible on a later [^68^Ga]Ga-PSMA-11 PET/CT or that new tumor foci would have appeared. Additionally, [^89^Zr]Zr-PSMA-617 PET/CT itself has the disadvantage of an ~ 2.5 times higher radiation exposure compared to conventional PSMA PET/CT [12]. However, in the present analysis, the substantial if not complete biochemical response seen in many patients given radiotherapy following positive [^89^Zr]Zr-PSMA-617 PET/CT suggests that this imaging procedure may provide outcome-altering information, which would appear to justify its incremental radiation exposure. Notably, with median PSA concentrations of 0.52 ng/mL, our cohort appeared to have early-stage BCR. At that crucial juncture, lesion localization may facilitate choice of appropriate treatments before recurrence becomes extensive. Unsurprisingly, in light of the nature of the disease, confirmation of scan accuracy and benefit of ^89^Zr-based imaging-guided therapy predominantly relied on biochemical response to treatment followed by post therapeutic imaging (in 7 patients) rather than on histopathology (in 1 patient); however, verification without histopathology is an usual methodology in - even prospective multicenter - PSMA PET studies [7, 20]. Histopathological confirmation of all [^89^Zr]Zr-PSMA-617 PET/CT findings of course would be optimal from a scientific point of view but would inflict unethical interventional burden on the patient.

## Conclusions

Our data in this large series provide further evidence that [^89^Zr]Zr-PSMA-617 PET/CT offers an impressive improvement of PSMA imaging to localize disease early in BCR. Disruptive increases of tumor-to-background ratio over time – in the order of 100 - allow tumor localization in a large majority of cases (positivity rate of 79% in our cohort), that were unidentified on conventional PSMA PET/CT, e.g., with [^68^Ga]Ga-PSMA-11 PET/CT.

## Data Availability

No datasets were generated or analysed during the current study.

## Abbreviations

^18^F: Fluorine-18
^68^Ga: Gallium-68
^89^Zr: Zirconium-89
ADT: Androgen deprivation therapy
CT: Computed tomography
max: Maximum
min: Minimum
MIP: Maximum intensity projection
PET: Positron emission tomography
p.i: Post-injection
PSA: Prostate-specific antigen
PSMA: Prostate-specific membrane antigen
SD: Standard deviation
SUV_max_: Maximum standardized uptake value
SUV_mean_: Mean standardized uptake value
TBR: Tumor-to-background ratio
TCR: Tumor-to-contralateral tissue ratio
TLR: Tumor-to-liver ratio
TMR: Tumor-to-gluteal muscle ratio
